# Fragment excision and triceps V-Y advanced reattachment using suture anchor for olecranon nonunion: A case report

**DOI:** 10.1097/MD.0000000000037700

**Published:** 2024-04-05

**Authors:** Tae-Yeong Kim, Jae-Shin Yang, Jung-Taek Hwang

**Affiliations:** a Department of Orthopaedic Surgery Hallym University Chuncheon Sacred Heart Hospital, Gangwon-do, Korea.

**Keywords:** nonunion, olecranon fracture, triceps reattachment

## Abstract

**Rationale:**

The nonunion of olecranon fractures is uncommon in simple fractures, and it is challenging to treat surgically due to the disruption of the anatomy of the elbow joint. There is limited literature on surgical options, and several factors to determine the treatment, including the amount and quality of bone stock, age, and degree of articular damage.

**Patient concerns:**

A 58-year-old man presented at the clinic with neglected olecranon fracture for 1 year (case 1). A 74-year-old man (case 2) presented with consistent pain and limited of motion after surgery for olecranon fracture.

**Diagnosis:**

Both patients were diagnosed with olecranon nonunion.

**Intervention:**

Both patients received the excision of nonunited fragment and reattaching with V-Y advancement of triceps.

**Outcomes:**

Range of motion and Mayo elbow performance score were improved after surgery.

**Lessons:**

This technique is useful in patients who cannot undergo other surgical options due to insufficient bone quality and elbow function, and it can lead to satisfactory outcomes with an acceptable range of motion and pain relief.

## 1. Introduction

Nonunion of olecranon fractures is uncommon in simple fractures, and most cases come from complex injuries, including fracture dislocation.^[1]^ It is hard to treat surgically because the anatomy of the elbow joint is destroyed with a sclerotic margin of the nonunion fragment, instability, and stiffness.^[2]^ There is little literature on this topic, and surgical options for nonunion of olecranon fractures depend on various considerations including age, the amount and quality of bone stock, and the degree of articular damage.^[1]^ Several methods have been previously mentioned. Painless fibrous nonunion with more than 90° range of motion (ROM) can be treated with no surgical treatment. Osteosynthesis with plates and bone grafts is the treatment of choice in young and active patients. Elbow arthroplasty should be used in elderly patients with severe arthritis. However, there are cases that do not fit into these categories, such as those with insufficient bone quality and elbow function to fix, and not much age to proceed with arthroplasty. For these cases, 1 technique that excises the nonunion fragment and reattaches the triceps tendon to the proximal ulna has been considered. Excision of the fragment with triceps advancement is used for older, lower-demand individuals with osteoporotic bone and more comminuted injuries.^[3]^ In this study, we excised the nonunion fracture fragment with a triceps flap and reattached the tendon to the proximal ulna with suture anchors.

## 2. Surgical technique

General anesthesia, regional anesthesia, or a combination can be used. After proper anesthesia, the patient is positioned supine on the operation table. The operative arm is prepped and draped in sterile fashion, and an upper extremity tourniquet is applied. A posterior incision just lateral to the tip of the olecranon is carried out using the previous incision. Sharp dissection is used to reflect full-thickness skin flaps to expose the dorsal ulna and triceps insertion. Granulation tissue is reflected at the nonunion site to facilitate visualization and cleaning of the site. The incision is extended proximally to the upper arm, and the triceps fascia is exposed. We cut the triceps tendon at the point 5 mm distal to the musculotendinous junction and excised the olecranon nonunion fragment from the triceps tendon. Using 2 biocork anchors (3.7 mm double-loaded Bio-Suture Tak anchor, Arthrex Inc. Naples, FL), the triceps tendon was reattached to the ulna using a Mason Allen suture, and the proximal triceps muscle was sutured to the V-Y advanced triceps tendon with ethibond #2-0 using a mattress type suture. The skin incisions are closed in a standard fashion, and a long-arm posterior splint is applied at 30° to 60° flexion. Antero-posterior and lateral radiographs are obtained postoperatively.

## 3. Case 1

**Figure 1.**
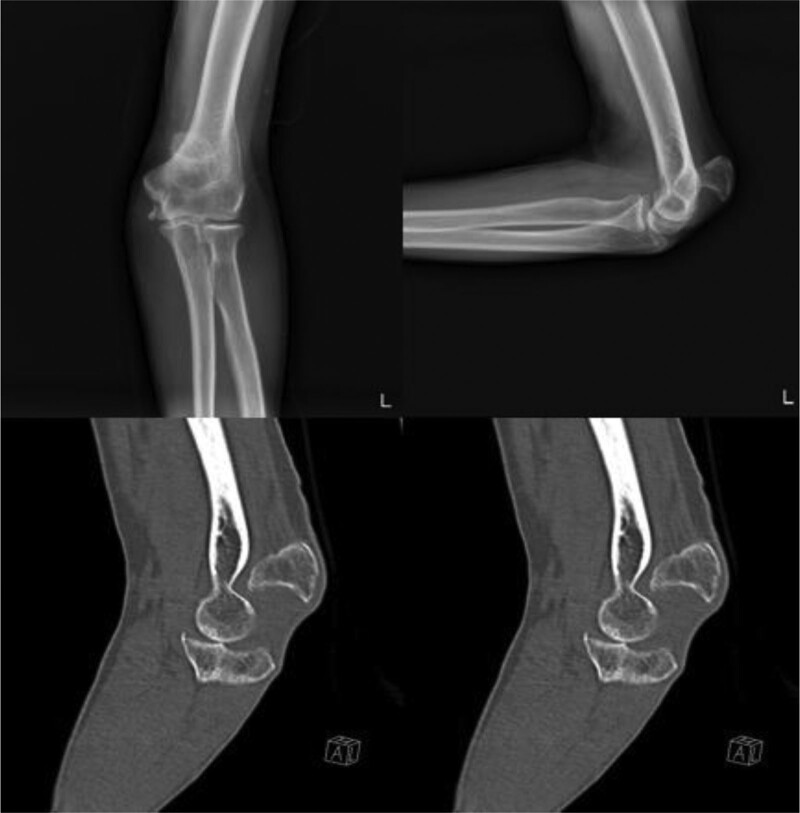
Preoperative X-ray and computed tomography showing olecranon nonunion fracture with separation.

**Figure 2.**
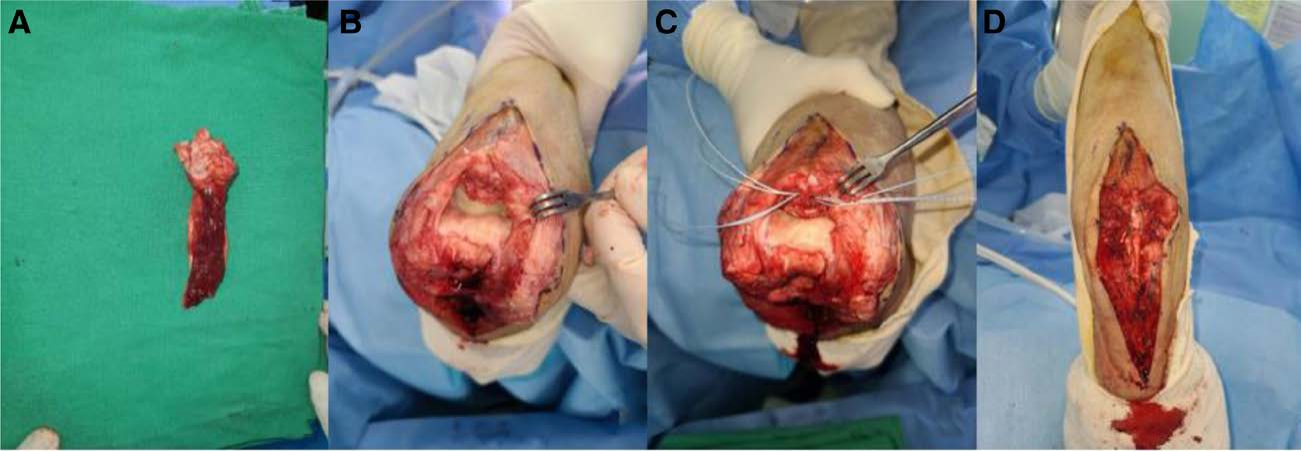
Intraoperative findings. (A) Excised fragment with triceps tendon flap. (B, C) Preparing for reattachment on proximal ulna. (D) Triceps reattachment was done with triceps V-Y advancement.

**Figure 3.**
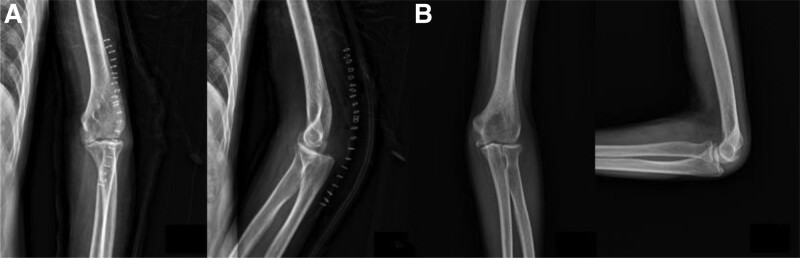
(A) Immediate postoperative X-ray. (B) After 3 mo follow-up X-ray.

A 58-year-old male visited our out-patient clinics via local clinics with left elbow pain. He fell down 1 year ago and did not visit any clinic until the out-patient visit. Tenderness was absent around olecranon area. Limited ROM was checked with 30° extension lag and 130° further flexion. Mayo elbow performance score was 40. During elbow motion, we can see skin dimpling on olecranon area that showed separation of bones. Both neurological and vascular examinations were normal. X-ray and computed tomography of the elbow showed the separation of nonunion fracture fragment of olecranon (Fig. 1). Fragment excision and triceps reattachment were performed. There were sclerotic surfaces on the nonunion fragment and tightness of the triceps tendon, so we proceeded with V-Y advancement (Fig. 2). After the operation, we applied a long-arm splint with 30° flexion, and immediate postoperative X-ray showed the absence of a bony fragment (Fig. 3A). Then, we changed the splint to a cast until postoperative day (POD) 4 weeks. After cast removal, we applied a 60° flexion splint and started intermittent 30° to 90° ROM exercise. On POD 6 weeks, we removed splint finally and proceed tolerable ROM exercise. On POD 15 weeks, there were no specific findings on X-ray (Fig. 3B), ROM was checked satisfactorily from 5° to 135°, and Mayo elbow performance score was 80. Afterward, the follow-up was lost.

## 4. Case 2

**Figure 4.**
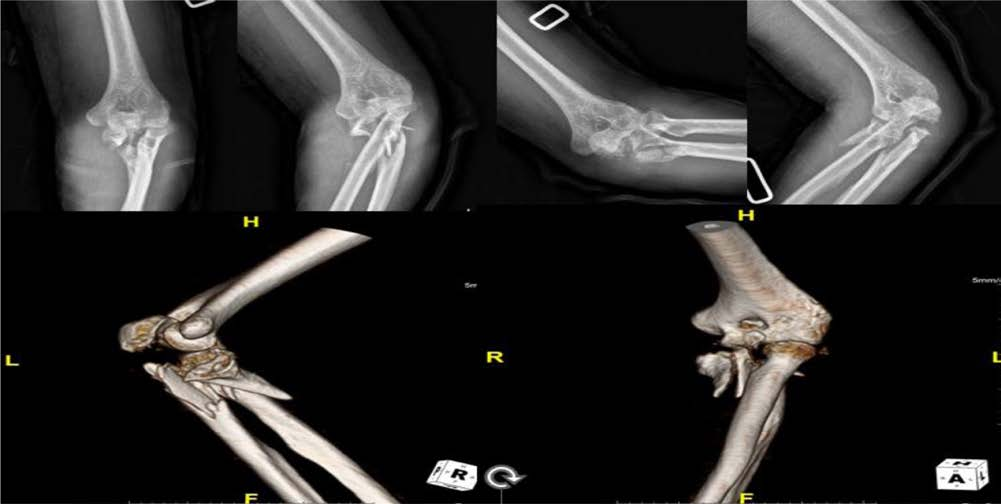
Preoperative X-ray and computed tomography showing comminuted fracture of proximal ulna including olecranon process and coronoid process.

**Figure 5.**
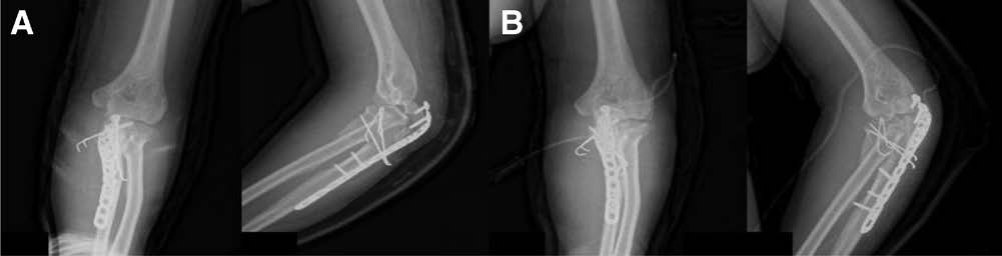
(A) Immediate postoperative X-ray after first operation. (B) Postoperative X-ray after revision.

**Figure 6.**
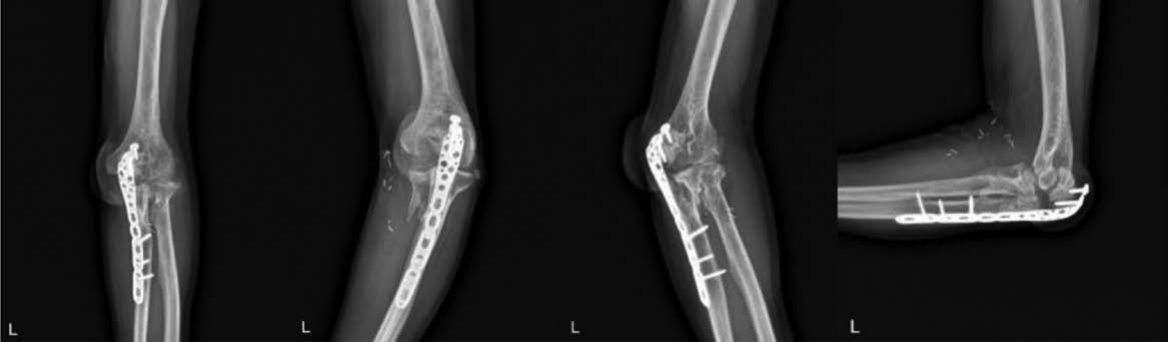
After 2 yr follow up, nonunion of olecranon can be seen in X-ray.

**Figure 7.**
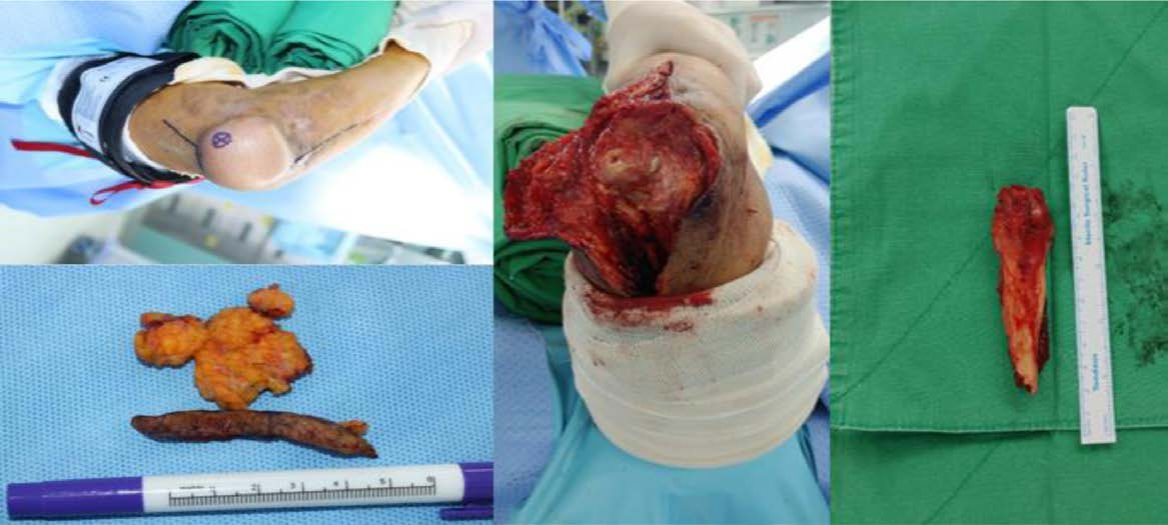
Intraoperative findings. Incision was extended from previous flap incision. Skin and tissue debulking were done. Nonunion fragment and triceps flap were excised.

**Figure 8.**
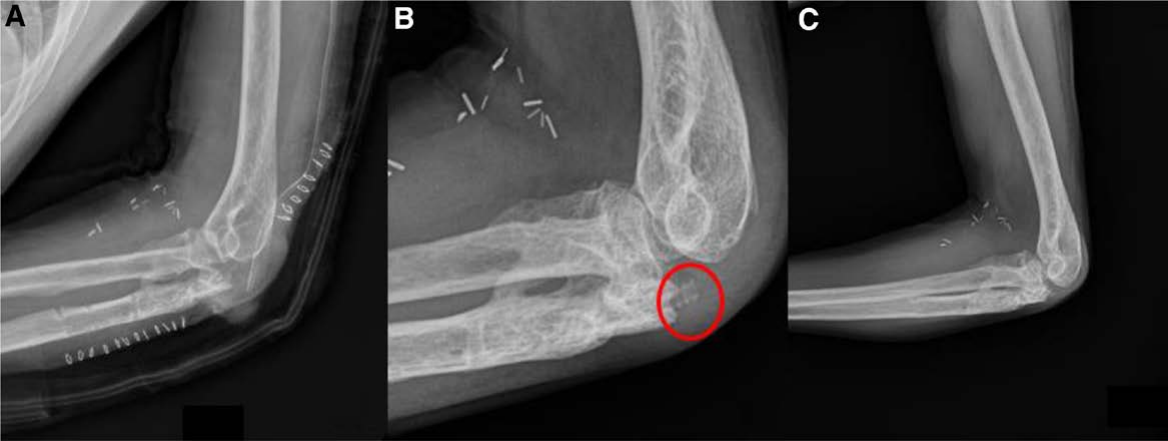
(A) Immediate postoperative X-ray. (B) POD 2 mo, protrusion of biocork screw was identified. (C) POD 7 mo, there was no more protrusion. POD = postoperative day.

A 74-year-old male was sent to our emergency department after motorcycle traffic accident. He had multiple traumas, including brain hemorrhage, hemothorax, and fractures of other parts, such as the superior and inferior ramus of the pelvis, the shaft of tibio-fibula. In this review, we focused on elbow fracture. The left elbow showed severe deformity, swelling. Both neurological and vascular examinations were normal. X-ray and computed tomography of the elbow showed a comminuted fracture of proximal ulna including olecranon process and coronoid process (Fig. 4). Eight days after the injury, an open reduction and internal fixation (ORIF) was performed a Synthes 2.7/3.5 mm variable angle locking compression plate (DePuy Synthes, West Chester, PA) and Kirschner wire for coronoid process (Fig. 5A). After 1 week, reduction loss was identified, and revisional ORIF was done (Fig. 5B). At POD 2 months, skin defect was present, and the plate was exposed, so vastus lateralis myocutaneous free flap reconstruction was done by a plastic surgeon. After a 2-year follow-up, X-ray and computed tomography showed nonunion of olecranon and elbow ROM was checked 40° to 100° (Fig. 6). So, we decided to perform fragment excision and triceps reattachment. Using previous incision, we did debulking previous flap lesion and removed olecranon plate and then unstable free fragment was identified. After excision of fragment and preparing for reattaching triceps tendon, reattachment was done using 2 biocork suture anchor and V-Y advancement of triceps tendon (Fig. 7). After operation, we applied long-arm splint with 45° flexion and immediate postoperative X-ray showed absences of bony fragment (Fig. 8A). Then, we changed splint to 90° flexion after 2 weeks. On POD 1 month, we removed splint finally and applied elbow brace with 30° to 90° ROM, educated limitation of active elbow extension. On POD 6 weeks, we changed brace ROM to 0° to 120° and continued limitation of extension. On POD 2 months, the forced extension limitation had been educated until POD 3 months, but the patient was noncompliant that he removed the brace and had driven motorcycle frequently as his own way. Follow-up X-ray showed half protrusion of biocork screw, but there was no symptom (Fig. 8B). On POD 7 months, there was no more protrusion of screw on X-ray (Fig. 8C), ROM was checked satisfactorily 30° to 120°, and Mayo elbow score was 75.

## 5. Discussion

Nonunion of the olecranon is one of the refractory complications of complex fractures with bone defect such as trans-olecranon fracture-dislocation and Monteggia fracture with posterior displacement^[4,5]^ and inappropriate stabilization of initial operation. Despite its rare incidence, nonunion of olecranon becomes a challenge for surgeon because it is hard to treat and can result in functional impairment and poor outcome.^[6]^ Currently, few studies have shown the treatment of this condition. Jakobi et al^[7]^ showed a treatment algorithm using autogenous bone grafting and additive compression plating those results in good outcomes and complete bony union. In this article, they emphasized anatomical reduction, but in complex olecranon fracture, like fracture dislocations, anatomy is commonly disrupted and unstable which makes reduction hard. In elderly, the risk of nonunion is increased due to osteoporosis and poor bone healing.^[8]^ Also, after treating nonunion, inferior outcome after revisional fixation in elderly have been shown in some studies.^[8–10]^

Therefore, we sought a method that can be used in nonunion or severe fractures. In olecranon fracture, fragment excision and triceps reattachment are an acceptable choice of treatment. This method has been reported to remove the possibility of joint disruption and malunion^[11]^ and metal problems.^[12]^ McKeever and Buck^[3]^ reported favorable results in those who underwent excision for acute fractures or nonunion of the olecranon fracture and showed that more than half had normal extension strength of the elbow. Gartsman et al^[12]^ compared excisions with internal fixation, reviewing 107 cases of olecranon fractures. Pain, function, strength, ROM, and the incidence of degenerative changes were similar between the 2 groups. We believe this method can be applied to the concept of treating nonunion. Due to its rare frequency and narrow indication, we have only used this method in 2 cases. Both cases showed satisfactory outcomes in elbow ROM and function, and radiologically, there was no further disruption of the joint. Compared to revisional reduction and fixation, this method is easy to perform. In revisional fixation, we should use autogenous bone graft so that an incision must be required on another side, especially the pelvis area, and an additional procedure like shaving off the surface of consolidation, anatomically reduction, firm fixation. It takes much more operation time than initial ORIF and requires an experienced surgeon and sufficient equipment. Fragment excision and triceps reattachment can be performed more simply than revision and take less operation time, averaging 43 minutes from incision to closure. Therefore, this method can be a considerable choice of treatment for nonunion of the olecranon. However, as there are only 2 cases using this method and no long-term outcomes, further study will be required to prove its availability.

## 6. Conclusion

Fragment excision and triceps V-Y advanced reattachment can be a considerable choice of treatment for nonunion of the olecranon especially in complex elbow trauma.

## Author contributions

**Conceptualization:** Tae-Yeong Kim, Jung-Taek Hwang.

**Writing—original draft:** Tae-Yeong Kim.

**Data curation:** Jae-Shin Yang.

**Formal analysis:** Jae-Shin Yang.

**Methodology:** Jung-Taek Hwang.

**Supervision:** Jung-Taek Hwang.
